# Characterizing chlorotriazine effects in cancer-relevant high-throughput screening assays

**DOI:** 10.3389/ftox.2025.1682439

**Published:** 2025-10-03

**Authors:** Agnes L. Karmaus, Alex Charlton

**Affiliations:** Syngenta Crop Protection, LLC, Greensboro, NC, United States

**Keywords:** chlorotriazine, atrazine, toxcast, Tox21, key characteristics of carcinogens

## Abstract

**Introduction:**

High-throughput screening (HTS) *in vitro* testing can be a powerful tool for evaluating chemicals across an abundance of mechanistic, targeted assay systems. This study reviewed HTS *in vitro* data for the systematic evaluation of endpoints relevant to carcinogenesis. To these means, we focused on assay endpoints from the ToxCast/Tox21 HTS program that have been mapped to Key Characteristics of Carcinogens (KCCs) to help focus our review on the ∼750 assay endpoints that have been previously identified as potentially informative for evaluating modes of action relevant to carcinogenesis.

**Methods:**

Data for ToxCast/Tox21 HTS assay endpoints were retrieved from the publicly accessible invitrodb v4.2 and reviewed for five chlorotriazine herbicides (atrazine, cyanazine, propazine, simazine, and terbuthylazine) to evaluate any indication of cancer-relevant bioactivity. More specifically, we present a workflow comprising the use of a focused assay endpoint inventory based on KCC attribution, integration of assay flags to identify robust bioactivity, and putting *in vitro* mechanistic insights into context with literature-based context for toxicokinetic considerations and *in vivo* evidence.

**Results and Discussion:**

There were common targets consistently identified as bioactive across the chemical class including induction of estrone levels and potential CAR/PXR activation. These findings were put in context by utilizing *in vivo* data and knowledge about atrazine to weigh the evidence. Though the ToxCast/Tox21 HTS mechanistic assays did not yield novel insights into chlorotriazine carcinogenicity, our workflow exemplifies how starting from mechanistic screening data and integrating apical endpoints can be conducted. By providing context to *in vitro* ToxCast/Tox21 data with toxicokinetics information and available *in vivo* study outcomes demonstrates how the HTS data and KCC framework can be applied to review a chemical class for carcinogenicity potential.

## 1 Introduction

*In vitro* testing systems offer valuable platforms for gaining insights into how chemicals might interact with biological targets at the cellular and molecular levels. High-throughput screening (HTS) testing of chemicals has generated tremendous datasets that have opened new avenues for gaining mechanistic insights, at the forefront of which are the ToxCast and Tox21 programs (Thomas et al., 2018). HTS *in vitro* assays yield snapshots of very specific interactions and effects, most of which require complementary data and integrated approaches to contextualize and appropriately interpret. For example, collecting results from orthologous assays–assays which evaluate the same biological target in completely independent assay systems and testing formats can bolster confidence in confirming bioactivity of chemicals. Furthermore, integrating multiple assay results across a defined pathway of events can be useful for understanding the exact interaction a chemical has with a biological target to elicit any disruptive effects (e.g., estrogen and androgen receptor pathway models; Judson et al., 2015; Kleinsteuer et al., 2017, respectively). Analyses that extrapolate from molecular and cellular responses to organ and organism level responses for regulatory decision making are challenging. The case study herein demonstrates an example of integrating *in vitro* HTS assay data and existing *in vivo* knowledge to facilitate understanding of toxicological mode of action.

Atrazine, a chlorotriazine herbicide and one of the most commonly applied pesticides globally, has been the subject of intense scrutiny and debate regarding its potential health impacts (Abarikwu et al., 2023). It has been tested widely across many mechanistic *in vitro* assay systems with a plethora of publicly available results, including from the ToxCast HTS program comprising ∼1,500 assay endpoints (Feshuk et al., 2023). However, the interpretation and contextualization of these mechanistic insights remains challenging.

Long term treatment with chlorotriazine herbicides is known to induce formation of mammary gland tumours in some strains of laboratory rats (Simpkins et al., 2011). The established toxicological mode of action for this specific female rat mammary tumor formation is the disruption of pulsatile release of gonadotropin releasing hormone (GnRH) from the hypothalamus which in turn attenuates the amplitude of the luteinizing hormone (LH) surge. The result of this hormonal disruption in rats is an increased duration of estrus and with prolactin levels which, over an extended period, elicits elevations in circulating estrogen and prolactin levels causing the formation of mammary adenocarcinomas and fibroadenomas. The formation of these tumors was determined to be the result of altered reproductive aging in some strains of laboratory rat and has no relevance for human risk or hazard assessment (Simpkins et al., 2011).

Despite a hypothesis for rat-specific mammary cancer etiology based on *in vivo* studies, the chlorotriazine chemical class as a whole requires further testing to supplement existing toxicological mode of action understanding, particularly for evaluating human response. This study seeks to organize primarily human-based *in vitro* mechanistic endpoints for the systematic evaluation of data relevant to carcinogenesis. The Key Characteristics of Carcinogens (KCC) framework was employed as it was explicitly developed as a tool to support the identification of potential carcinogenic agents (Smith et al., 2016; Smith et al., 2020). It is important to note that chemical carcinogens may have the potential to exhibit one or more of the KCCs which include the ability to: induce genomic instability, alter DNA repair mechanisms, cause epigenetic alterations, induce oxidative stress, trigger chronic inflammation, affect cell proliferation and death, disrupt cell energy metabolism, or modulate receptor-mediated effects. Whilst the KCC are not suitable for outright cancer prediction (Becker et al., 2017), the KCCs may provide a useful framework for the organization of mechanistic evidence. The KCC framework has been criticized as not comprehensively reflecting the required weight of evidence needed for making a deterministic assessment of cancer potential (Borgert, 2023). More specifically, the KCC approach does not encompass all information needed for predicting carcinogenic response in laboratory animals or in human; the omitted factors include quantitative thresholds for progression through an adverse outcome pathway, tissue specificity of response, *in vivo* kinetic factors, and biological organization. Yet, associating assay endpoints with KCCs has been proposed to support the organization of ToxCast and Tox21 HTS mechanistic evidence for use in cancer hazard identification (Samet et al., 2020). Thus, by broadly examining the bioactivity of chlorotriazine herbicides in ToxCast with a KCC focus, we propose that potential modes of action for (human) carcinogenesis not previously identified for this chemical class could be found.

## 2 Materials and methods

### 2.1 Chlorotriazine chemicals evaluated

The focus of this data review was chlorotriazine herbicides evaluated in the Tox21/ToxCast HTS program. The chlorotriazine herbicides are part of a wider class of herbicides which function through the inhibition of photosystem II (PSII). PSII inhibitors include compounds from a range of chemical areas, including ureas, amides, and triazines (including both chlorinated and non-chlorinated members). Chlorotriazines have a well-characterized toxicological mode of action in mammals, which is not shared by other members of the PSII group. Due to this well-understood mechanism of toxicity, the chlorotriazines were of most interest for ouranalysis. Five chlorotriazines were thus in scope: atrazine (CASRN 1912-24-9; DTXSID9020112), cyanazine (CASRN 21725-46-2; DTXSID1023990), propazine (CASRN 139-40-2; DTXSID3021196), simazine (CASRN 122-34-9; DTXSID4021268), and terbuthylazine (CASRN 5915-41-3; DTXSID4027608). All chemicals were procured and distributed for testing to assay vendors by the United States Environmental Protection Agency as part of the ToxCast HTS program or were procured explicitly for the Tox21 HTS program (Richard et al., 2016; Richard et al., 2021). No new testing was conducted as part of this study.

### 2.2 High-throughput screening data retrieval

ToxCast and Tox21 high-throughput screening (HTS; Dix et al., 2007; Judson et al., 2010; Tice et al., 2013) data were retrieved directly from the MySQL invitrodb database (Feshuk et al., 2023), version 4.2 (U.S. Environmental Protection Agency, 2025). Within this database, all assay endpoints’ raw data and analysis results from the ToxCast Pipeline (tcpl) concentration-response and hit calling algorithm are stored. All analyses were performed using R Statistical Software (v4.3.0; R Core Team, 2021). Relevant ToxCast data were retrieved using the *tcpl* package (v3.2.1; Feshuk et al., 2023; Filer, 2025). Briefly, level 5 (concentration-response modeling and hit calling) and level 6 (flagging) data for all assay endpoints were retrieved utilizing the *tcplLoadData* function. Data were subset to the representative sample/replicate using the *tcplSubsetChid* function to simplify the dataset in a supported and reproducible manner. All code for data retrieval and processing are available in Supplementary File 1.

### 2.3 HTS data interpretation workflow

HTS data were reviewed to ensure only biologically interpretable and robust active assay endpoints were considered. Briefly, the retrieved level 5 hit call values were reviewed to identify putatively active endpoints with a hit call ≥0.9. This hit call cutoff has generally been accepted as it has been demonstrated to yield consistent results compared to historical interpretations for ToxCast assay hit calling (Feshuk et al., 2023). More importantly, requiring hit call ≥0.9 is most likely to ensure that a concentration-dependent, biologically interpretable effect was detected based on the three parameters contributing to the hit call calculation being met: 1. the best fitting mathematical model for the concentration-response data is not driven by noise; 2. at least one testing concentration’s experimental replicates’ median exceeds the cutoff set for the assay (a cutoff calculated per assay endpoint to separate baseline response from biologically interpretable response); 3. the modeled concentration-response curve’s top exceeds the cutoff (Feshuk et al., 2023). Putatively active assay endpoints having four or more level 6 cautionary flags, a fit category of 36 (indicating AC50 is extrapolated as being less than or equal to the minimum concentration tested), or a hit call of −1 (indicating a non-biologically relevant fitting direction) were considered low confidence and dismissed.

The AC50 concentrations from the remaining putatively active concentration-response results were reviewed to ensure that the potency of putative bioactivity was more potent than any confounding overt toxicity from cytotoxicity evaluations. Concurrent cytotoxicity assay endpoint AC50’s per assay technology were evaluated where available, else the chemical’s computed “cytotoxicity burst” concentration (Judson et al., 2016) was used as a representative estimate of the concentration at which the chemical elicits non-specific cell stress/cytotoxicity effects.

The remaining active assay endpoints represent the most robust bioactivity (with high confidence hit call metrics, minimal curve-fitting flags or extrapolation outside testing concentrations, and not confounded by cytotoxicity) for which the AC50 concentrations were retrieved to serve as a metric representing the potency for analyses herein. For a list of all retrieved data, level 6 cautionary flags, AC50 values, and hit calls, see Supplementary File 2.

### 2.4 Cancer-relevant assay inventory

The ToxCast and Tox21 HTS assay inventory is annotated in multiple annotation schema to support interpretation of the assay endpoints. For example, the invitrodb database contains tables that provide technological and “intended target” biological and gene annotations (U.S. Environmental Protection Agency, 2025; Feshuk et al., 2023), the Integrated Chemical Environment (ICE; U.S. National Toxicology Program, 2025) contains complementary annotations that provide “mode of action” and “mechanistic target” in a toxicological context, and the Tox21 Tripod (US NIH/NCATS 2025) contains detailed information about the Tox21 assay protocols. With a firm understanding of the assay platforms, endpoints, biological significance, and toxicological interpretation, it is possible to narrow down which assays are relevant for any given context of use. To these ends, prior efforts to associate the Key Characteristics of Carcinogens (KCC; Smith et al., 2016) to HTS assays have been published including suggested guidance (Guyton et al., 2018) on use and assay mappings conducted by the IARC Monographs Workgroup (Chiu et al., 2018) and more recently by an international collaboration seeking to update and refine the original mappings (generously provided by Cuomo et al., manuscript in prep). Herein, we leveraged the most recent updated mapping which aligns with assay names in the current version of invitrodb database v4.2.

## 3 Results

### 3.1 Chlorotriazine bioactivity in cancer-relevant assays

ToxCast/Tox21 HTS assay endpoints annotated to the KCCs, testing outcomes were retrieved for chlorotriazines focusing on atrazine, cyanazine, propazine, simazine, and terbuthylazine (Table 1). Overall, the chlorotriazines were tested in ∼600–700 assay endpoints out of a total of ∼1,500 assay endpoints in the ToxCast inventory. When limiting to assays that have been mapped to KCCs, the number of assays in which chlorotriazines have been tested is ∼350 (Table 1). It is important to note that the identify of the assay endpoints tested are not the same across the chlorotriazines, though most were in common. All five chlorotriazines were evaluated in 587 assay endpoints when considering all of ToxCast, of which 323 assay endpoints were mapped to KCCs.

**TABLE 1:** Summary of chlorotriazines tested and assay endpoints active in ToxCast.

	Assay endpoints tested	Active assay endpoints	Active assay endpoints with <4 flags
Atrazine	ToxCast: 761	ToxCast: 57 (7.5%)	ToxCast: 54
	KCC Mapped: 390	KCC Mapped: 41 (10.5%)	KCC Mapped: 38
Cyanazine	ToxCast: 679	ToxCast: 41 (6.0%)	ToxCast: 36
	KCC Mapped: 363	KCC Mapped: 33 (9.1%)	KCC Mapped: 29
Propazine	ToxCast: 664	ToxCast: 46 (6.9%)	ToxCast: 45
	KCC Mapped: 355	KCC Mapped: 31 (8.7%)	KCC Mapped: 30
Simazine	ToxCast: 695	ToxCast: 21 (3.0%)	ToxCast: 21
	KCC Mapped: 377	KCC Mapped: 18 (4.8%)	KCC Mapped: 18
Terbuthylazine	ToxCast: 603	ToxCast: 47 (7.8%)	ToxCast: 44
	KCC Mapped: 332	KCC Mapped: 37 (11.1%)	KCC Mapped: 35

^a^ percentage calculated as the number of active assay endpoint out of number tested assay endpoints.

The chlorotriazines each had an active rate of 3%–8% across ToxCast (Table 1); simazine showed the lowest active assay endpoint incidence at 3% (21 active calls across 695 assay endpoints tested) while terbuthylazine showed the highest at 7.8% (47 active calls across 603 assay endpoints). When focusing on assay endpoints mapped to KCCs (799 assay endpoints from ToxCast were considered attributable/mapped to KCCs for the current study; Supplementary File 2) the number of relevant assay endpoints tested drops to ∼350 per chlorotriazine and the percentage of active endpoints lies between 5% and 11% (Table 1).

Just as not all chemicals are tested in every assay endpoint, not all KCCs are well covered by ToxCast assay endpoints (Table 2). To evaluate where chlorotriazine-elicited active assay endpoints occur within the context of KCCs, active assay endpoint hit calls were also summarized per KCC, per chlorotriazine (Table 2). It is not surprising, given the abundance of nuclear receptor transactivation assays that KCC8 (modulation of receptor-mediated effects) comprised the majority of mapped and active endpoints. KCC10 (alteration of cell proliferation, cell death, or nutrient supply) also had a few active assays for most chlorotriazines. Very low, one or two, active endpoints were found for KCC6 (induction of chronic inflammation) and KCC7 (immunosuppression) among chlorotriazines despite 60+ assay endpoints for these KCCs. Near complete inactivity was seen for KCC2 (genotoxic), KCC3 (altering DNA repair/causing genomic instability), KCC4 (induction of epigenetic alterations), and KCC5 (induction of oxidative stress).

**TABLE 2:** Summary of active assay endpoints per KCC.

	KCC2	KCC3	KCC4	KCC5	KCC6	KCC7	KCC8	KCC10
	Is genotoxic	Alters DNA repair, causes genomic instability	Induces epigenetic alterations	Induces oxidative stress	Induces chronic inflammation	Is immunosuppressive	Modulates receptor-mediated effects	Alters cell proliferation, cell death, or cell nutrient supply
Total Mapped^a^	22	31	13	62	156	67	369	215
Atrazine					1	2	31	5
Cyanazine					2	1	22	5
Propazine				2	2	1	23	2
Simazine					1	1	16	
Terbuthylazine					2		29	5

A given assay endpoint may be mapped to more than one KCC (and thus be represented twice in any row).

^a^ Total mapped assay endpoints summarize assay annotations and not testing (e.g., not every assay mapped to KCCs, may have chlorotriazines tested).

**FIGURE 1:**
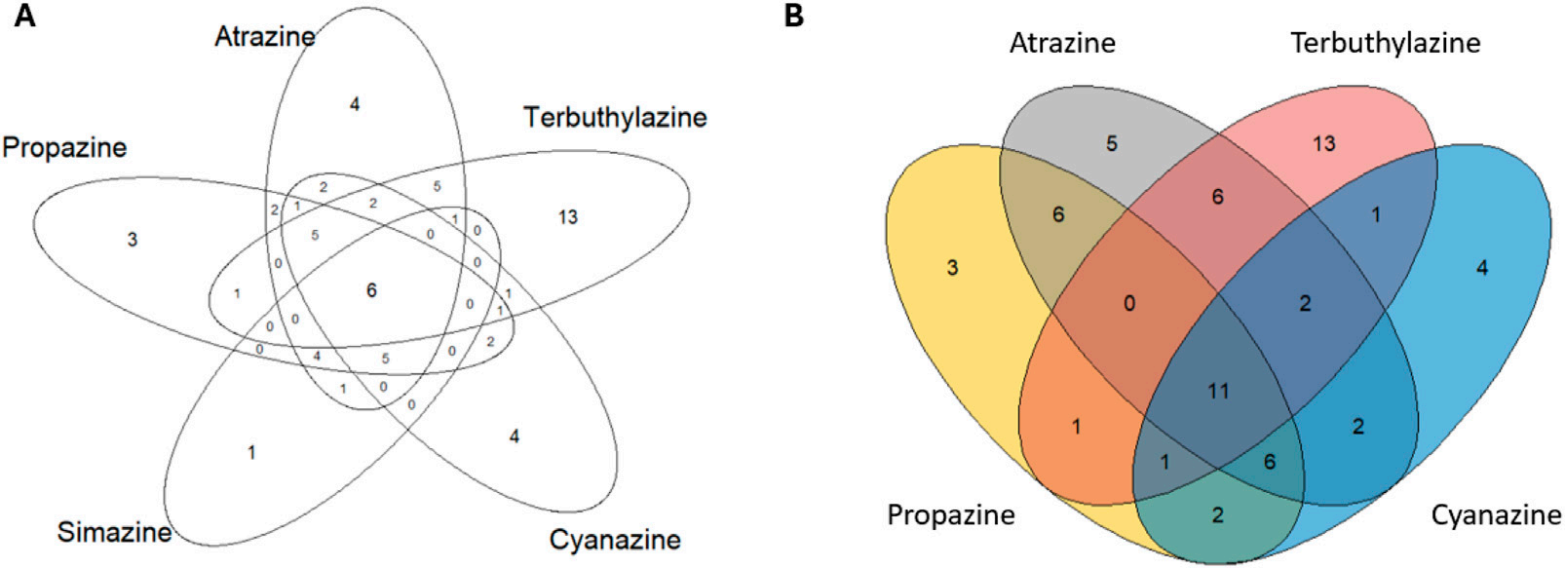
Venn diagrams comparing the identity of active assay endpoints across the evaluated chlorotriazines, **(A)** For all five chlorotriazines (atrazine, cyanazine, propazine, simazine, and terbuthylazine), **(B)** Four chlorotriazines comparison, excluding simazine as it had the lowest testing and active hit call rate. The assay endpoint IDs (aeids) for assay endpoints in every segment of both Venn diagrams are provided in Supplementary File 2.

To gauge how consistent results were across the chlorotriazine class, active assay endpoints were compared using a Venn diagram (Figure 1). While some differences are driven by testing (e.g., overlap would not be possible if not all chemicals were tested in all assay endpoints), it is informative to see which assay endpoints unanimously yield consistent calls across all chlorotriazines tested. There are six assay endpoints in which all chlorotriazines were tested and active; when removing simazine (the lowest testing rate and bioactivity rate) the overlap among the remaining chlorotriazines identified 11 consistently active assay endpoints (Supplementary File 2).

**FIGURE 2:**
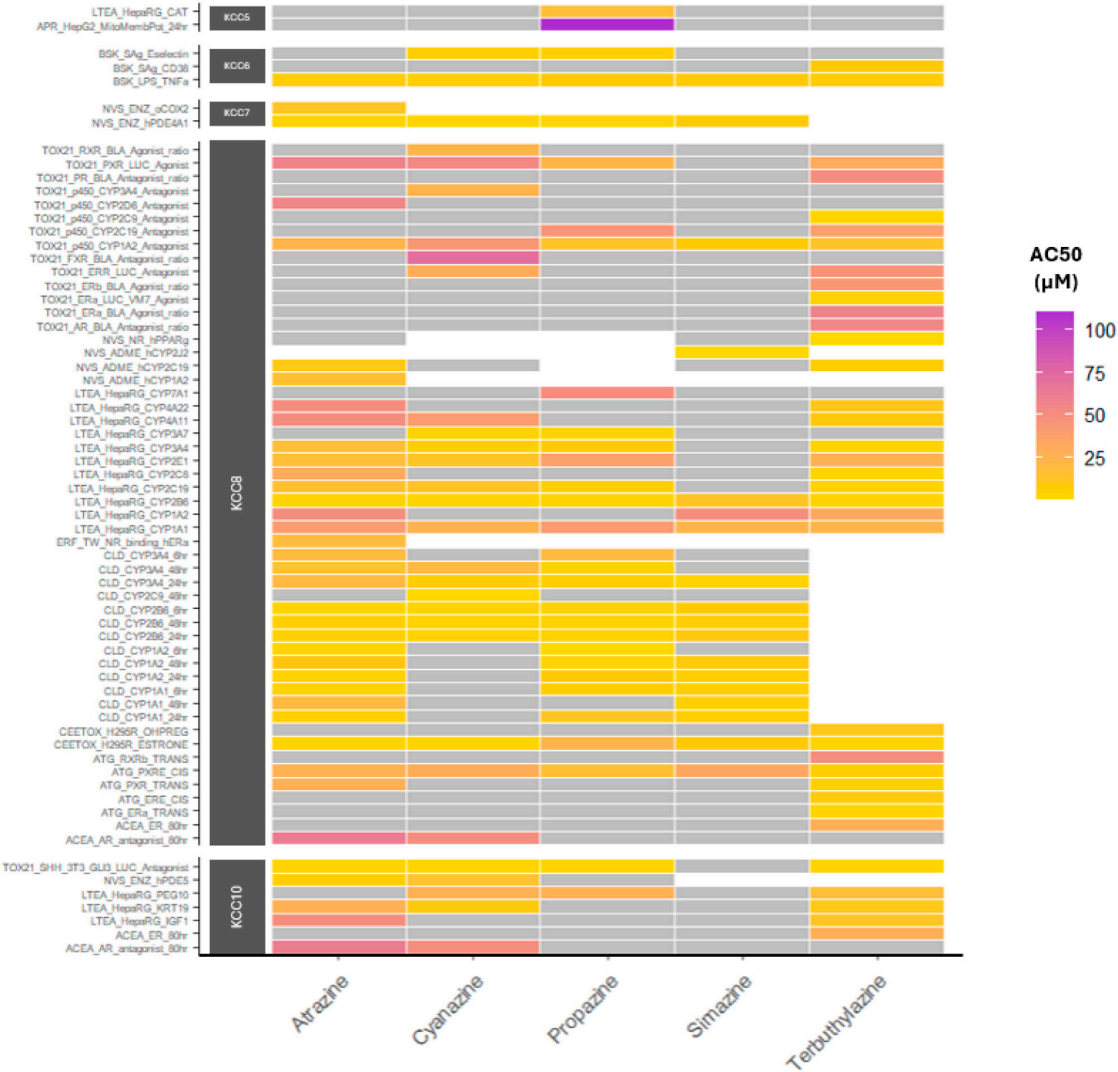
Heatmap of AC50 potency values for active assay endpoints. The AC50 (µM concentration at which half maximal effect is observed) was plotted as a heatmap for all chlorotriazines to review the potency of effects across active endpoints, grouped by KCC with assays in alphabetical order. To simplify visualization, only assays in which at least one chlorotriazine was active are plotted; a complete heatmap figure with all assays in which chlorotriazines were tested is provided in Supplementary File 3. White indicates not tested, grey indicates inactive calls and low-confidence actives (e.g., for the purpose of plotting, active calls with four or more flags were considered inactive).

The relative potencies of the active assay endpoints for all chlorotriazines was also reviewed (Figure 2). This evaluation identifies consistency across the chlorotriazines regarding endpoints that are potently active, for example the CellsDirect gene expression assays (assay endpoint names starting CLD) in which mRNA levels for key metabolic enzymes such as CYP1A1, CYP2B6 and CYP3A4 are induced by all chlorotriazines with AC50 values lower than 20 µM. Figure 2 visualizes all assays in which at least one chlorotriazine was active; Supplementary File 3 contains an expanded heatmap in which all assays with at least one chlorotriazine tested are plotted (this larger Supplementary Figure is useful for visualizing the abundance of consistent inactive assay endpoints).

### 3.2 Chlorotriazine effects on CAR/PXR assay endpoints

Among the most consistent effects noted from all chlorotriazines across the KCC-relevant HTS assay endpoints was the induction of metabolic enzyme gene expression (see Supplementary File 2 for identities of assay endpoints from Venn comparison in Figure 1). The LifeTech Expression Analysis (LTEA) battery of endpoints in ToxCast originate from a multiplex assay quantifying the mRNA levels for 96 relevant transcripts from human hepatocyte HepaRG cells to inform on transcription factor activation (Franzosa et al., 2021). Among the assay endpoints commonly active across at least four of the five evaluated chlorotriazines were the LTEA endpoints for CYP1A1, CYP2B6, CYP2C19, CYP2E1, and CYP3A4 transcripts. Additionally, the CLD assay endpoints quantifying CYP2B6 and CYP3A4 at multiple time points were also commonly induced by four chlorotriazines. These endpoints are related to KCC8 “modulates receptor mediated effects”, as these transcripts’ induction would be mediated by a transcription factor. Most common among these transcripts is mediation by CAR/PXR.

**FIGURE 3:**
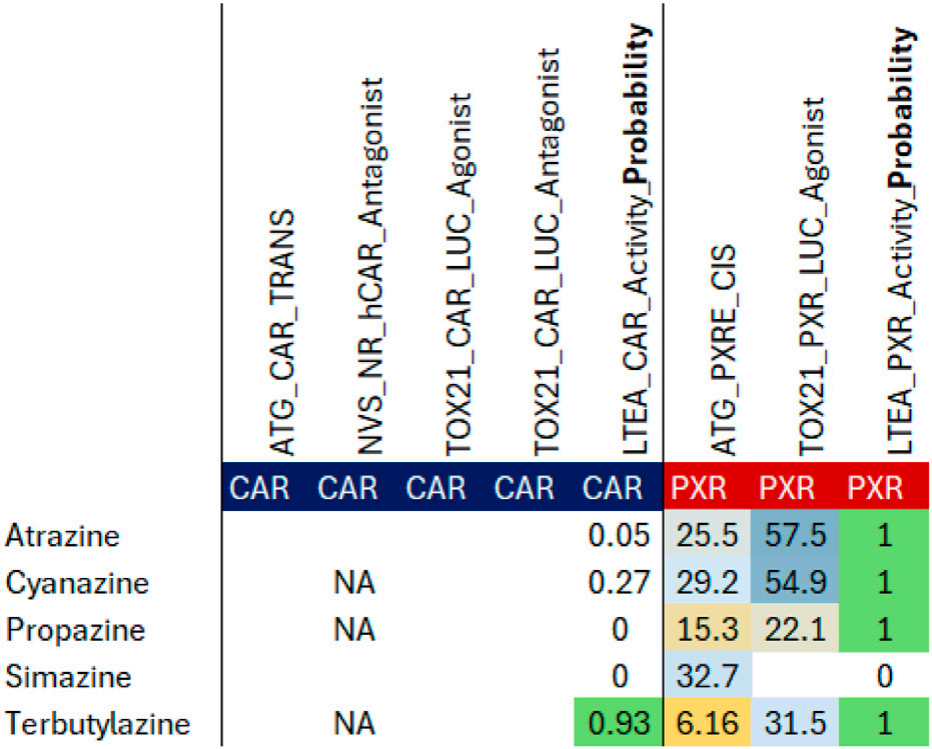
Evaluation of CAR/PXR Assay Endpoints for all Chlorotriazines. Values represent AC50 (µM) where assay endpoint was active (hitcall >0.9) except for the LTEA probabilities (derived from Franzosa et al., 2021; Supplementary Material, tab 6) where values represent probabilities. White indicates inactive; NA indicates not applicable where chemical was not tested in the assay. Color scale for assay endpoints runs from yellow (most potently active: lowest AC50) to blue (least potent: highest AC50); green indicates significant probability for transcription factor activation (Franzosa et al., 2021).

The consistent detection of human hepatocyte transcripts regulated by CAR/PXR warrants further review. To this end, we assessed additional analyses to bolster the biological interpretation of these gene expression findings. We reviewed the modeling conducted with the LTEA endpoints to aggregate mRNA expression data for predicting transcription factor activation (Figure 3). Terbuthylazine was the only chlorotriazine that yielded a likely probability (0.93) for CAR activation based on modeling LTEA gene expression data, while assays evaluating CAR transactivation for all other members of the class were negative. Conversely, when reviewing PXR transactivation assays and LTEA modeling results, atrazine, cyanazine, propazine, and terbuthylazine all yielded 1.0 (highest possible probability) for predicted PXR activation. These four chlorotriazines also yielded active calls in PXR transactivation assays (ATG_PXRE_CIS and TOX21_PXR_LUC_Agonist assay endpoints; Figure 3). In fact, the Attagene PXR transactivation assay (ATG_PXRE_CIS) yielded active calls for all five chlorotriazines evaluated all with AC50 values ranging from 6 to 32 µM (Figure 3).

### 3.3 Phosphodiesterase, aromatase, and steroidogenesis

Inhibition of phosphodiesterase (PDE) enzyme isoforms has been proposed as a potential alternative mode of action for rat mammary tumours observed following long term treatment with atrazine (Simpkins et al., 2011). In this putative mechanism inhibition of PDE enzymes results in intracellular increases in cyclic AMP (cAMP) and subsequent increases in the expression of the aromatase enzyme. Increased aromatase expression may result in a greater rate of conversion of androgens to estrogens, and a higher lifetime estrogen burden resulting in mitogenesis of the mammary tissue and ultimately neoplasia.

ToxCast includes assay endpoint evaluating the inhibition of two human PDE isoforms, PDE4A1 and PDE5. These are cell-free enzymatic reporter loss of function assays from NovaScreen (NVS); endpoints are named NVS_ENZ_hPDE4A1 and NVS_ENZ_hPDE5, for PDE4A1 and PDE5, respectively (Sipes et al., 2013). Terbuthylazine has not been tested in either assay and will be excluded from this discussion. Atrazine and Cyanazine were active in both PDE4A1 and PDE5 assay endpoints. Simazine was only tested in the PDE4A1 assay where it was active for PDE4A1 inhibition with an AC50 of 8.46 µM. Notably, propazine was the only examined compound to produce an inactive response in a PDE assay, with an active PDE4A1 response and an inactive PDE5 response. Where compounds were active in PDE4A1 or PDE5 assays a marginally greater level of potency was observed against PDE4A1 than PDE5 (AC50 ranges for active responses of 0.39–1.2 µM and 4.96–13.61 µM, for PDE4A1 and PDE5 respectively).

The ToxCast assays for aromatase are not expression, but rather enzyme inhibition assays. None of the chlorotriazines elicited effects on aromatase activity, which is not surprising given there is no evidence that atrazine or any of the chlorotriazines inhibit aromatase thus the assay format may not be appropriate to evaluate this potential mode of action.

Since aromatase is responsible for the metabolism of androgens to estrogens, we reviewed the ToxCast multiparametric high-throughput H295R steroidogenesis assay (HT-H295R) focusing on the estrogen and testosterone hormone level endpoints. The HT-H295R assay detected a consistent increase in estrone levels, but not estradiol, for all chlorotriazines (Table 3). Manual review of these concentration-response plots showed that atrazine marginally increased estrone levels just above the assay cutoff (an assay endpoint specific parameter distinguishing baseline from biologically interpretable response; (Feshuk et al., 2023). Whilst estradiol levels were also marginally elevated, it remained below the activity threshold thus resulting in an inactive hit call for all chlorotriazines. We retrieved the AC50 for even the negative estradiol hit calls to compare potency (as all concentration-response data are modeled no matter the ultimate hit call), revealing that the increases in estrone and estradiol had similar AC50 values of 0.97 µM and 1.06 µM with atrazine treatment, respectively. The concentration range of this effect is thus comparable to the potency level at which chlorotriazines inhibited the PDE enzymes.

**TABLE 3:** Summary of chlorotriazines in phosphodiesterase, aromatase, and steroidogenesis assay endpoints.

	Assay target	Assay endpoint name	Hit call	Hit call value	Flags	AC50 (µM)
Atrazine	PDE4A1	NVS_ENZ_hPDE4A1	TRUE	1.00	13	1.22
	PDE5	NVS_ENZ_hPDE5	TRUE	0.98	9; 13; 17	4.96
	Aromatase	NVS_ADME_hCYP19A1	FALSE	0.00	8; 13	NR
	Aromatase	TOX21_Aromatase_LUC_Antagonist	FALSE	0.00	20	NR
	Steroidogenesis	CEETOX_H295R_ESTRADIOL	FALSE	0.00	8; 9; 11	NR
	Steroidogenesis	CEETOX_H295R_ESTRONE	TRUE	1.00	NA	1.35
	Steroidogenesis	CEETOX_H295R_TESTO	FALSE	0.00	NA	NR
Cyanazine	PDE4A1	NVS_ENZ_hPDE4A1	TRUE	1.00	13	0.39
	PDE5	NVS_ENZ_hPDE5	TRUE	0.99	13; 17	13.61
	Aromatase	NVS_ADME_hCYP19A1	NT	NT	NT	NT
	Aromatase	TOX21_Aromatase_LUC_Antagonist	FALSE	0.01	NA	NR
	Steroidogenesis	CEETOX_H295R_ESTRADIOL	FALSE	0.00	8; 9	NR
	Steroidogenesis	CEETOX_H295R_ESTRONE	TRUE	0.99	NA	1.11
	Steroidogenesis	CEETOX_H295R_TESTO	FALSE	0.00	NA	NR
Propazine	PDE4A1	NVS_ENZ_hPDE4A1	TRUE	1.00	13	1.00
	PDE5	NVS_ENZ_hPDE5	FALSE	0.00	9; 13	NR
	Aromatase	NVS_ADME_hCYP19A1	NT	NT	NT	NT
	Aromatase	TOX21_Aromatase_LUC_Antagonist	FALSE	0.00	20	NR
	Steroidogenesis	CEETOX_H295R_ESTRADIOL	FALSE	0.00	9; 11	NR
	Steroidogenesis	CEETOX_H295R_ESTRONE	TRUE	1.00	NA	23.14
	Steroidogenesis	CEETOX_H295R_TESTO	FALSE	0.00	NA	NR
Simazine	PDE4A1	NVS_ENZ_hPDE4A1	TRUE	0.99	13	8.46
	PDE4	NVS_ENZ_hPDE5	NT	NT	NT	NT
	Aromatase	NVS_ADME_hCYP19A1	FALSE	0.00	9; 13	NR
	Aromatase	TOX21_Aromatase_LUC_Antagonist	FALSE	0.00	20	NR
	Steroidogenesis	CEETOX_H295R_ESTRADIOL	FALSE	0.23	8; 9; 11	NR
	Steroidogenesis	CEETOX_H295R_ESTRONE	TRUE	1.00	NA	8.07
	Steroidogenesis	CEETOX_H295R_TESTO	FALSE	0.00	9	NR
Terbuthylazine	PDE4A1	NVS_ENZ_hPDE4A1	NT	NT	NT	NT
	PDE5	NVS_ENZ_hPDE5	NT	NT	NT	NT
	Aromatase	NVS_ADME_hCYP19A1	NT	NT	NT	NT
	Aromatase	TOX21_Aromatase_LUC_Antagonist	FALSE	0.16	11	NR
	Steroidogenesis	CEETOX_H295R_ESTRADIOL	FALSE	0.00	8; 9; 11	NR
	Steroidogenesis	CEETOX_H295R_ESTRONE	TRUE	0.98	9; 11	1.51
	Steroidogenesis	CEETOX_H295R_TESTO	FALSE	0.00	NA	NR

NA, not applicable (e.g., there were no flags reported).

NT, not tested.

NR, not reported (e.g., if hit call is false, no AC50 is reported herein as it is not biologically interpretable).

## 4 Discussion

Herein, we have reviewed publicly available high-throughput *in vitro* testing data for chlorotriazines with a focus on gaining insight on potential bioactivity relevant to carcinogenic modes of action. While a proposed hormone-mediated mode of action for atrazine-elicited tumors in rats has been proposed (Simpkins et al., 2011), the molecular initiating event for this pathway has not been established and the majority of processes in this pathway (comprising circulating GnRH and LH hormone level changes) are not assessed by the targeted assays among the ToxCast HTS inventory. Thus, to further cast a broad net, we sought to anchor the current evaluation to the KCC framework allowing for HTS data review in an organized and informed manner.

The first focus for the evaluation of chemical agents when considering carcinogenicity is frequently the ability to damage genetic material or to cause mutations as genetic alterations are fundamental to the process of cancer development. Approximately two thirds of chemicals identified as carcinogens in rodent bioassays are genotoxicants, whilst 90% of known human carcinogens are genotoxic (Hernández et al., 2009). This difference may be attributable to the potential for non-genotoxic agents to exhibit exaggerated or unrepresentative responses in rodent cancer bioassays conducted at very high doses that do not reflect human exposure to the agents of interest (Ames and Gold, 1990). Genotoxicity testing is a central component of regulatory compliance in the agrochemical industry, and regulatory bodies such as the European Food Safety Authority (EFSA) and the United States Environmental Protection Agency (US EPA), require a battery of *in vitro* and *in vivo* genotoxicity tests to evaluate the potential of agrochemicals to induce genetic damage (European Food Safety Authority Scientific Committee, 2011a; U.S. Environmental Protection Agency, 2007). This typically includes a combination of tests to detect gene mutations, chromosomal aberrations and aneuploidy, and DNA damage (Kirkland et al., 2011).

The ToxCast high-throughput assay inventory does contain endpoints intended to assess the genotoxic potential of test compounds; these assays primarily focus on key events in the genotoxicity adverse outcome pathway, such as DNA damage response, cell cycle arrest, and p53 activation (Knight et al., 2009). These assays provide insights into potential genotoxic mechanisms of action, however they do not fully replicate the endpoints assessed in regulatory genotoxicity testing batteries. Missing endpoints include direct assessment of gene mutations, chromosomal aberrations, and micronucleus formation. In addition, the assays on ToxCast do not include metabolic activation systems, such as S9 incubation, and this limits their ability to detect pro-genotoxicants (Wambaugh et al., 2018). The absence of an effect on genotoxicity and oxidative stress linked assays in ToxCast across the chlorotriazine class is consistent with previous conclusions in this area: comprehensive regulatory reviews of atrazine (U.S. Environmental Protection Agency, 2018a), terbuthylazine (European Food Safety Authority, 2011b), simazine (U.S. Environmental Protection Agency, 2018b), propazine (U.S. Environmental Protection Agency, 2018c), and cyanazine (California Environmental Protection Agency, 1997) have established the non-genotoxic nature of compounds in the chlorotriazine class.

The most common target identified across all five chlorotriazines from this review of ToxCast assay endpoints suggested that most chlorotriazines may act as PXR agonists, characterized by bioactivity in PXR transactivation assays and the induction of PXR-regulated CYP transcripts in human liver cells from the CLD and LTEA *in vitro* gene expression assays. However, gene expression alone does not provide insight on functional outcome or biological effect, so further evidence was sought to contextualize the relevance of these findings. Upon reviewing the literature, we identified only one study in which *in vivo* data from hepatic microsomes yielded a detectable induction of CYP2B in Fisher rats intraperitoneally exposed to atrazine for 24 h (Islam et al., 2002). The *in vivo* data for atrazine administration to laboratory animals is typically confined to characterization of minor increases in relative liver weight (normalized to animal body weight) with a consistent absence of any histological or clinical chemistry correlates. Overall, atrazine is not considered a liver carcinogen in any laboratory test species (Joint FAO/WHO Meeting on Pesticide Residues, 2007), indicating that, whilst this class may activate the PXR nuclear receptor, the overall evidence suggests that this activation does not correlate to any downstream biological changes *in vivo*.

Finally, we considered the data which could putatively be evaluated offering insight on the hormone-mediated mammary gland carcinogenesis in response to atrazine exposure documented in Sprague Dawley rats (Stevens et al., 1999). While the established mode of action for this *in vivo* finding is the disruption of pulsatile GnRH release from the hypothalamus attenuating LH surge amplitude (Simpkins et al., 2011), a proposed alternative hypothesis postulates that elevations in estrogen levels, as a result of increased expression of the aromatase enzyme, may elicit mammary carcinogenesis across mammalian species, including humans (Fan et al., 2007; Bulun et al., 2005). However, atrazine is not a mammary gland carcinogen in ovariectomized female Sprague Dawley rats (Stevens et al., 1999), suggesting that endogenous estrogen formation is required and that induction of aromatase alone may not be sufficient for the development of mammary gland tumors. Furthermore, atrazine does not result in mammary tumors in Fischer-344 rats (Wetzel et al., 1994; Thakur et al., 1998), in CD1 mice (Stevens et al., 1999), nor in humans based on weight of evidence evaluations (Simpkins et al., 2011; U.S. Environmental Protection Agency, 2018a). It should be acknowledged that rodents and humans have different aromatase regulation and expression, especially in peripheral tissues (Zhao et al., 2016). As such, we have reviewed the effects of chlorotriazines on phosphodiesterases (PDEs), aromatase enzyme expression, and sex hormones levels in ToxCast human-based assays to thoroughly consider the proposed alternative mode of action for the observed Sprague Dawley rat mammary tumors.

To comprehensively evaluate the proposed alternative mode of action, we started with PDE enzymes which exhibit widespread tissue distribution and play a role in regulating intracellular levels of cyclic nucleotides, particularly cyclic adenosine monophosphate (cAMP) and cyclic guanosine monophosphate (cGMP; Conti and Beavo, 2007; Díaz-Cruz et al., 2005). Inhibition of PDE4 can lead to enhanced aromatase expression through the activation of protein kinase A and subsequent phosphorylation of cAMP response element-binding protein (Díaz-Cruz et al., 2005). Similarly, PDE5 inhibition may indirectly affect aromatase expression by modulating nitric oxide (NO) signaling, which can influence cAMP levels through crosstalk between cGMP and cAMP pathways (Tsai et al., 2011). Aromatase is a critical enzyme in estrogen biosynthesis, catalyzing the conversion of androgens to estrogens (Simpson et al., 2002; Mayr and Montminy, 2001); the aromatase gene (CYP19A1) contains CRE in its promoter region, which is one of the major promoters regulating aromatase expression in breast cancer tissues (Bulun et al., 2005). Upregulation of aromatase expression may increase local estrogen production and thereby promote proliferation of estrogen-sensitive tissues (Simpson et al., 2002). This relationship is complex, with the effects of PDE inhibition on aromatase activity varying depending on tissue context and the PDE isoform targeted (Keravis and Lugnier, 2012).

Previous studies have demonstrated the inhibitory effects of atrazine on PDE4 and in turn downstream increases in aromatase expression *in vitro* (Sanderson et al., 2000; Fan et al., 2007; Higley et al., 2010). Despite the potential link between inhibition of PDEs and aromatase induction, the ToxCast aromatase inhibition assays did not show any response to chlorotriazine exposure. The Tox21 aromatase inhibition assay does not assess the induction of the aromatase gene’s expression, but rather the enzyme’s catalytic activity, and as such an absence of an effect of atrazine in this assay system does not conflict with available studies indicating that atrazine increases the expression of aromatase *in vitro*. The steroidogenesis HT H295R assay endpoints for estrogens (estradiol and estrone) both yielded marginal trending increases in hormone levels, with potencies comparable to those noted for PDE inhibition. While these results may appear supportive of the hypothesized alternative mode of action, other factors such as toxicokinetics regarding chlorotriazine’s rapid metabolism, need to also be considered.

*In vivo*, atrazine is rapidly metabolized, with a plasma half-life of 4 h, to form three major chlorometabolites: deethylatrazine (DEA), deisopropylatrazine (DIA), and diaminochlorotriazine (DACT) (U.S. Environmental Protection Agency, 2018a). This rapid metabolism has been documented for all chlorotriazines: simazine (U.S. Environmental Protection Agency, 2018b), propazine (U.S. Environmental Protection Agency, 2018c), terbuthylazine (European Food Safety Authority, 2011b), and cyanazine (California Environmental Protection Agency, 1997). The chlorometabolites exhibit lower *in vitro* potency for inhibition of PDE when compared to atrazine (Roberge et al., 2004). In fact, DIA was assayed in ToxCast and was active in PDE4A1 inhibition and estrone induction assay endpoints (Supplementary File 4). Consistent with previous findings (Roberge et al., 2004), the potency of DIA-mediated PDE4A1 inhibition was less than atrazine (e.g., the AC50 for the NVS_ENZ_hPDE4A1 assay endpoint informing on PDE4A1 inhibition for atrazine was 1.22 µM for DIA was 25 µM). A very comparable order of magnitude difference in potency was noted for the induction of estrone (CEETOX_H295R_estrone assay endpoint for atrazine had a potency of 1.35 µM whereas DIA was 24.56 µM). Chlorometabolites have also been evaluated for effects on aromatase activity in H295R human adrenocortical cells demonstrating that DACT does not induce aromatase activity while DIA elicits much lower efficacy compared to atrazine, and DEA may be the most efficacious of chlorometabolites regarding aromatase activity (Sanderson et al., 1999; Sanderson et al., 2001). Lower potency of DACT relative to atrazine for aromatase expression and estrogen induction has also been previously published (Tinfo et al., 2011). ToxCast screening did not include evaluation of DEA or DACT in any assays. Further testing of chlorometabolites would be required to confirm relative potency against the chlorotriazines. Given the current understanding of the ADME for chlorotriazines indicating rapid *in vivo* metabolism to chlorometabolites, ToxCast data should be integrated with *in vivo* kinetic data for safety decision making. This would ultimately allow for proper contextualization of not only the toxicokinetic considerations but also the relative differences in potency we have noted between chlorotriazines and chlorometabolites.

The presented evaluation of chlorotriazine ToxCast bioactivity data through the lens of the KCC framework yielded three potential toxicological modes of action warranting investigation: (1) the potential for this chemical class to exhibit damage to DNA which may drive tumor formation, (2) the ability to act as hepatic nuclear receptor agonists, and (3) effects on PDE enzymes and estrogen synthesis. By leveraging the well-studied atrazine with a wealth of *in vivo* rodent data and the breadth of HTS testing across predominantly human-based test systems and targets in ToxCast, the analyses herein integrated mechanistic insights anchored by KCCs and previously proposed hypothesis that could be relevant for humans.

This paper presents a worked example of how targeted *in vitro* screening data representing molecular interactions and focused biochemistry can be integrated into complex biological interpretation, in this example carcinogenesis. The KCC framework was used as an organizing framework for the interpretation of ToxCast data across a chemical class, accommodating variation in the identity of assays tested across the class. We leveraged established linkages of KCCs to ToxCast assays, assessed the reliability of individual assay hit calls using assay flags, integrating *in vitro* lines of evidence with available toxicokinetics, ADME data, and *in vivo* study data to assess the feasibility of mechanistic hypotheses for chlorotriazines likelihood to elicit carcinogenesis in humans. This approach demonstrates that contextualizing *in vitro* data, considering each assay’s context of use to ensure appropriate interpretation, integrating toxicokinetics, and leveraging multiple chemicals from a chemical class can help build weight of evidence to characterize chemical effects.

## Data Availability

The original contributions presented in the study are included in the article/Supplementary Material, further inquiries can be directed to the corresponding author.
