# Supplementary material for: Characterizing chlorotriazine effects in cancer-relevant high-throughput screening assays
Source: Front Toxicol. 2025 Oct 3;7:1682439. doi: 10.3389/ftox.2025.1682439 (PMC12531184; doi:10.3389/ftox.2025.1682439)
Supplement: Supplementary file 1 [file Supplementaryfile1.docx]

#=============================================================================#

# File Name: Chlorotriazine KCC ToxCast.R #

# Original Creator: Agnes Karmaus #

# Last Edited : Agnes Karmaus #

# Contact Information: agnes.karmaus@syngenta.com #

# Date Created: 2025-03-31 #

# Date Last Modified: 2025-08-07 #

# Description: Workflow for processing Tox21/ToxCast data retrieved from #

# invitrodb v4.2 to evaluate chlorotriazines in KCC-mapped assays #

# Required Packages: tcpl, data.table, ggvenn, venn, gplots, ggplot2 #

#==============================================================================#

library(tcpl)

library(data.table)

library(ggvenn)

library(venn)

library(gplots)

library(ggplot2)

#==============================================================================#

#### Retrieve dataset from invitrodb ###########################################

## dataset is retrieved from direct connection to invitrodb

## must ensure tcpl is configured to connect to local invitrodb using tcplconf()

## retrieve chemical inventory's ToxCast/Tox21 sample IDs

ch <- tcplLoadChem()

ch2 <- ch[casn %in% c("1912-24-9", "21725-46-2", "139-40-2",

"122-34-9", "5915-41-3")]

sp <- tcplQuery("SELECT * FROM sample")

## retrieve assay data from invitrodb

t5 <- tcplLoadData(5)

t5s <- tcplSubsetChid(t5)

t6 <- tcplLoadData(6)

t6sub <- t6[ , list(m5id, m6id, mc6_mthd_id, flag)]

t6w <- dcast.data.table(t6sub, m5id~mc6_mthd_id, value.var = "mc6_mthd_id")

setkey(t5s, m5id)

setkey(t6w, m5id)

dat56 <- t6w[t5s]

cols <- c("5", "6", "7", "8", "9", "10", "11", "13", "14", "15", "17", "18", "19", "20")

dat56[ , flags := Reduce(function(...) paste(..., sep = ";"), .SD[, mget(cols)])]

dat56[ , flags := gsub("\\NA;", "", flags)]

dat56[ , flags := gsub(";NA", "", flags)]

dat56[ , c(cols) := NULL]

## limit retrieved ToxCast/Tox21 data to chemical inventory

tri <- dat56[spid %in% ch2$spid]

## limit retrieved ToxCast/Tox21 data to KCC-mapped assays

kcc_assays <- fread("Triazine KCC\\KCCbyNUMBERS_long.csv")

tri_kcc <- tri[aeid %in% unique(kcc_assays$aeid)]

setkey(tri_kcc, spid)

setkey(sp, spid)

setkey(ch2, spid)

test <- sp[tri_kcc]

test <- ch2[tri_kcc]

test[ , c("i.chid", "i.casn", "i.chnm",

"i.dsstox_substance_id", "i.code") := NULL]

## add invitrodb assay details to data

a <- tcplQuery("SELECT * FROM assay_component_endpoint")

setkey(a, aeid)

setkey(test, aeid)

tkcc <- a[test]

## add KCC mapping

tkcc[aeid %in% kcc_assays[KCC == 2, aeid], kcc2 := TRUE]

tkcc[aeid %in% kcc_assays[KCC == 3, aeid], kcc3 := TRUE]

tkcc[aeid %in% kcc_assays[KCC == 4, aeid], kcc4 := TRUE]

tkcc[aeid %in% kcc_assays[KCC == 5, aeid], kcc5 := TRUE]

tkcc[aeid %in% kcc_assays[KCC == 6, aeid], kcc6 := TRUE]

tkcc[aeid %in% kcc_assays[KCC == 7, aeid], kcc7 := TRUE]

tkcc[aeid %in% kcc_assays[KCC == 8, aeid], kcc8 := TRUE]

tkcc[aeid %in% kcc_assays[KCC == 10, aeid], kcc10 := TRUE]

## curated hit call

tkcc[hitc == FALSE, hitc_curated := 0]

tkcc[hitc == TRUE, hitc_curated := 1]

tkcc[fitc == 36, hitc_curated := 2]

tkcc[nflg > 3, hitc_curated := 2]

# tkcc[hitc_curated == 2, list(aeid, chnm, nflg, flags)]

## write csv of all retrieved data

write.csv(tri, file = "Supplementary Table X.csv", row.names = FALSE)

## write csv of all KCC mapped assay data

write.csv(tkcc, file = "Supplementary Table Y.csv", row.names = FALSE)

#==============================================================================#

#### Table Generation ##########################################################

## Table 1: counts for chlorotriazine specific testing/activity in all ToxCast

tri[ , length(unique(aeid)), by = chnm]

tri[hitc == TRUE, length(unique(aeid)), by = chnm]

tri[hitc == TRUE & nflg < 4, length(unique(aeid)), by = chnm]

## Table 1: counts for chlorotriazine specific testing/activity in KCC assays

tkcc[ , length(unique(aeid)), by = chnm]

tkcc[hitc == TRUE, length(unique(aeid)), by = chnm]

tkcc[hitc_curated == 1, length(unique(aeid)), by = chnm]

## Table 2: mapped assay count per KCC

kcc_assays[ , .N, by = KCC]

## Table 2: active assay endpoint per chlorotriazine per KCC

## summary counts for which KCC have testing/activity

tkcc[ , length(unique(aeid)), by = list(kcc2, kcc3, kcc5, kcc6, kcc7, kcc8, kcc10)]

tkcc[ , length(unique(aeid)), by = list(chnm, kcc2, kcc3, kcc4, kcc5, kcc6, kcc7, kcc8, kcc10)]

tkcc[hitc_curated == 1, length(unique(aeid)),by = list(chnm, kcc2, kcc3, kcc4, kcc5, kcc6, kcc7, kcc8, kcc10)]

#==============================================================================#

#### Figure Generation #########################################################

## venn diagram: tested assay endpoints across all chlorotriazines

x <- list(

Propazine = tri[casn == "139-40-2", aeid],

Atrazine = tri[casn == "1912-24-9", aeid],

Terbuthylazine = tri[casn == "5915-41-3", aeid],

Cyanazine = tri[casn == "21725-46-2", aeid],

Simazine = tri[casn == "122-34-9", aeid]

)

venn(x)

attributes(gplots::venn(x, show.plot = FALSE))$intersections

## venn diagram: KCC-mapped tested assay endpoints across all chlorotriazines

x <- list(

Propazine = tkcc[casn == "139-40-2", aeid],

Atrazine = tkcc[casn == "1912-24-9", aeid],

Terbuthylazine = tkcc[casn == "5915-41-3", aeid],

Cyanazine = tkcc[casn == "21725-46-2", aeid],

Simazine = tkcc[casn == "122-34-9", aeid]

)

venn(x)

attributes(gplots::venn(x, show.plot = FALSE))$intersections

## venn diagram: active KCC-mapped endpoints across all chlorotriazines

x <- list(

Propazine = tkcc[casn == "139-40-2" & hitc_curated == 1, aeid],

Atrazine = tkcc[casn == "1912-24-9" & hitc_curated == 1, aeid],

Terbuthylazine = tkcc[casn == "5915-41-3" & hitc_curated == 1, aeid],

Cyanazine = tkcc[casn == "21725-46-2" & hitc_curated == 1, aeid],

Simazine = tkcc[casn == "122-34-9" & hitc_curated == 1, aeid]

)

venn(x)

## print aeid identities per section for supplementary file

attributes(gplots::venn(x, show.plot = FALSE))$intersections

## venn diagram: active KCC-mapped endpoints for only four chlorotriazines

ggvenn(x,

fill_color = c("#EFC000FF", "#868686FF", "#CD534CFF", "#0073C2FF"),

stroke_size = 0.5, set_name_size = 4, show_percentage = FALSE

)

## heatmap for assays with at least one active, by KCC

temp <- tkcc[ , list(chnm, aenm, hitc_curated, ac50,kcc2, kcc3, kcc4,

kcc5, kcc6, kcc7, kcc8, kcc10)]

temp[grep("chloro", chnm), chnm := "Propazine"]

temp[chnm == "Terbutylazine", chnm := "Terbuthylazine"]

temp_plot <- melt.data.table(temp, id.vars = 1:4, measure.vars = 5:12,

variable.name = "KCC")

temp_plot <- temp_plot[value == TRUE, ]

temp_plot[hitc_curated != 1, ac50 := NA]

plot_assays <- unique(temp_plot[hitc_curated == 1, aenm])

ggplot(temp_plot[aenm %in% plot_assays], aes(x = chnm, y = aenm, fill = ac50)) +

geom_tile(color="white", size = 0.25) +

# geom_text(aes(label = n)) +

# theme_bw() +

facet_grid(KCC~., scales="free_y", space="free_y") +

theme(panel.grid.major = element_blank(), panel.grid.minor = element_blank(),

panel.background = element_blank(), axis.line = element_line(colour = "black"),

axis.text.x = element_text(angle = 45, vjust = 0.95, hjust=1),

axis.text.y = element_text(size = 5)) +

scale_fill_gradient(low="gold", high="darkorchid", na.value="grey")

## heatmap for all assays with at least one chlorotriazine tested, by KCC

temp <- tkcc[ , list(chnm, aenm, hitc_curated, ac50,kcc2, kcc3, kcc4,

kcc5, kcc6, kcc7, kcc8, kcc10)]

temp[grep("chloro", chnm), chnm := "Propazine"]

temp[chnm == "Terbutylazine", chnm := "Terbuthylazine"]

temp_plot <- melt.data.table(temp, id.vars = 1:4, measure.vars = 5:12,

variable.name = "KCC")

temp_plot <- temp_plot[value == TRUE, ]

temp_plot[hitc_curated != 1, ac50 := NA]

#save as PDF for supplementary file

pdf("hetmap_all.pdf", width = 8, height = 25)

ggplot(temp_plot, aes(x = chnm, y = aenm, fill = ac50)) +

geom_tile(color="white", size = 0.25) +

# geom_text(aes(label = n)) +

# theme_bw() +

facet_grid(KCC~., scales="free_y", space="free_y") +

theme(panel.grid.major = element_blank(), panel.grid.minor = element_blank(),

panel.background = element_blank(), axis.line = element_line(colour = "black"),

axis.text.x = element_text(angle = 45, vjust = 0.95, hjust=1),

axis.text.y = element_text(size = 5)) +

scale_fill_gradient(low="gold", high="darkorchid", na.value="grey")

dev.off()
