# Supplementary figures and images for: Characterizing chlorotriazine effects in cancer-relevant high-throughput screening assays
Source: Front Toxicol. 2025 Oct 3;7:1682439. doi: 10.3389/ftox.2025.1682439 (PMC12531184; doi:10.3389/ftox.2025.1682439)

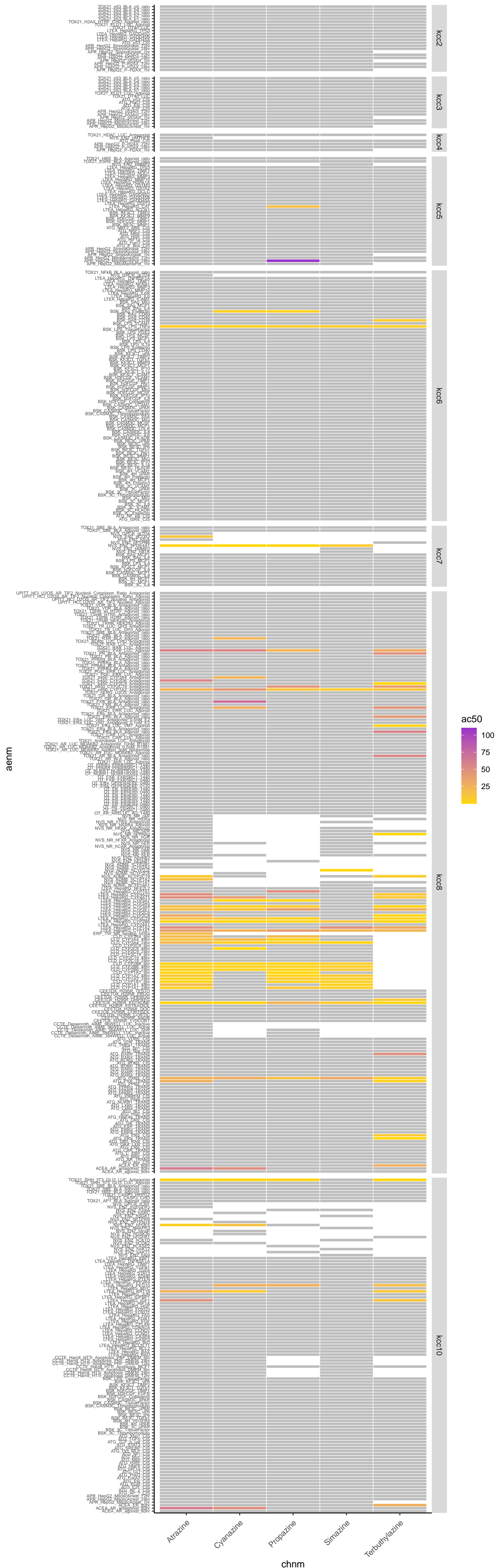

Supplement: Supplementary file 2 [file Supplementaryfile2.pdf]
