# Supplementary material for: Characterizing chlorotriazine effects in cancer-relevant high-throughput screening assays
Source: Front Toxicol. 2025 Oct 3;7:1682439. doi: 10.3389/ftox.2025.1682439 (PMC12531184; doi:10.3389/ftox.2025.1682439)
Supplement: Supplementary file 3 [file Supplementaryfile4.docx]

Comparison of atrazine and one of its chlorometabolites, deisopropylatrazine (DIA), was conducted to evaluate relative profile and potency of active cancer-relevant assay endpoints. Simazine was also included for further context as it also shares DIA as a major chlorometabolite.

**A** **B**


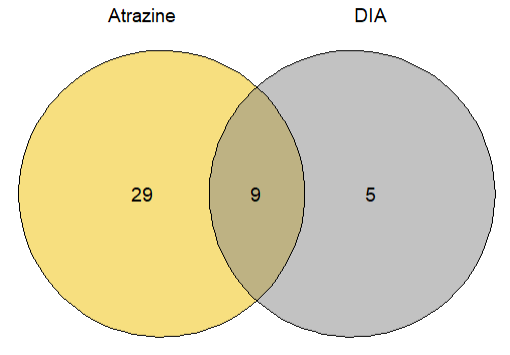

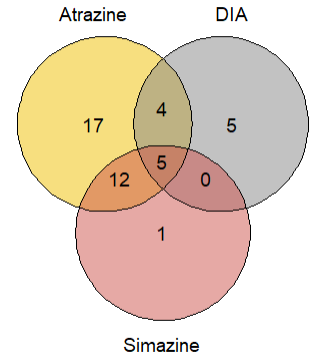


**Figure S1: Venn Diagram comparison of active assay endpoints**. (A) Comparison between atrazine and its chlorometabolite, DIA. (B) Simazine and atrazine were compared to DIA since they are both metabolized into DIA. Active assay endpoints represented in the sums for this figure were the result of filtering wherein robust actives were identified by omitting any actives that had 4 or more flags or a fit category of 36.

**Table S1: Identity of assay endpoints from Venn comparisons**


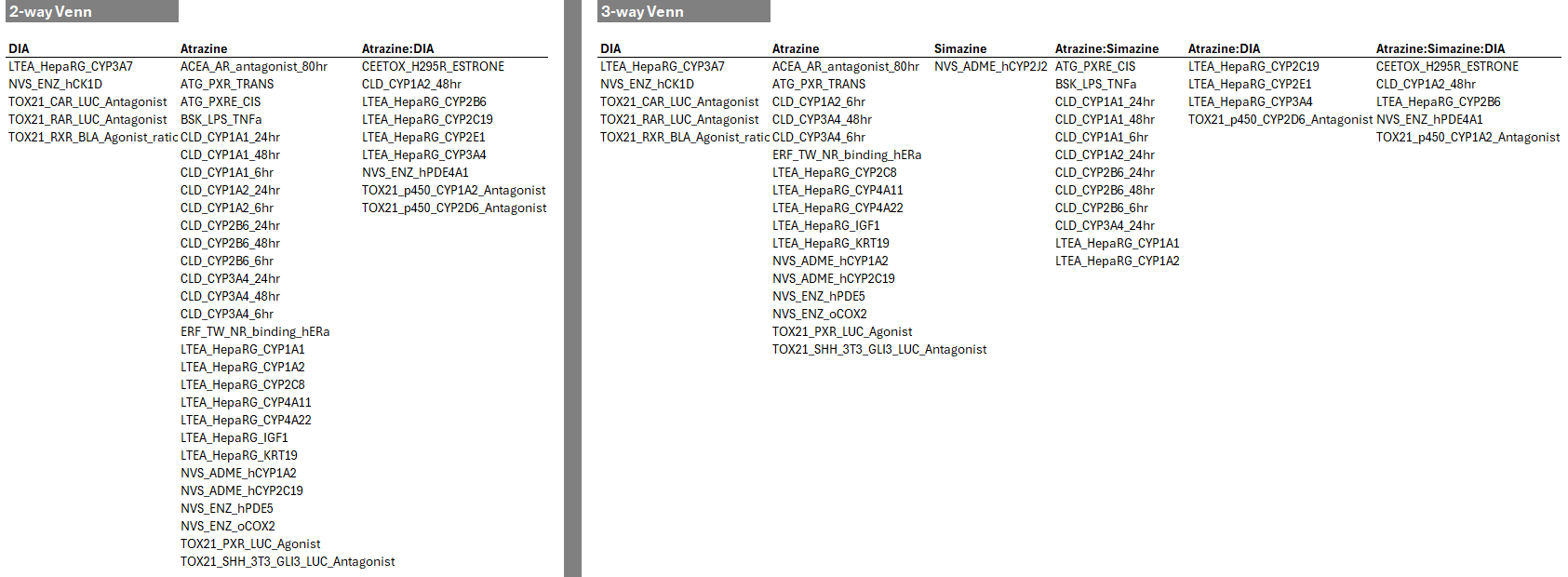


**Table S2: Potency of active assay endpoints in common Atrazine, Simazine, and DIA**

| Assay Endpoint name | atrazine | simazine | dia |
| --- | --- | --- | --- |
| CEETOX_H295R_ESTRONE | 1.35 | 8.07 | 24.56 |
| CLD_CYP1A2_48hr | 10.15 | 9 | 4 |
| LTEA_HepaRG_CYP2B6 | 2.34 | 11.33 | 6.87 |
| LTEA_HepaRG_CYP2C19 | 15.24 | NA | 6.65 |
| LTEA_HepaRG_CYP2E1 | 16.63 | NA | 50.00 |
| LTEA_HepaRG_CYP3A4 | 15.68 | NA | 3.18 |
| NVS_ENZ_hPDE4A1 | 1.22 | 8.46 | 25.00 |
| TOX21_p450_CYP1A2_Antagonist | 23.32 | 7.71 | 33.35 |
| TOX21_p450_CYP2D6_Antagonist | 55.8 | NA | 94.91 |

Note: all values represent AC50 in µM for active endpoints. NA: not active
